# Identification of Novel Smoothened Ligands Using Structure-Based Docking

**DOI:** 10.1371/journal.pone.0160365

**Published:** 2016-08-04

**Authors:** Celine Lacroix, Inbar Fish, Hayarpi Torosyan, Pranavan Parathaman, John J. Irwin, Brian K. Shoichet, Stephane Angers

**Affiliations:** 1 Department of Pharmaceutical Sciences, Leslie Dan Faculty of Pharmacy, University of Toronto, Toronto, Ontario, Canada; 2 Department of Pharmaceutical Chemistry, University of California San Francisco, San Francisco, California, United States of America; 3 Department of Biochemistry and Molecular Biology, George S. Wise Faculty of Life Sciences, Tel-Aviv University, Ramat Aviv, Israel; 4 Department of Biochemistry, Faculty of Medicine, University of Toronto, Toronto, Ontario, Canada; Indiana University School of Medicine, UNITED STATES

## Abstract

The seven transmembrane protein Smoothened is required for Hedgehog signaling during embryonic development and adult tissue homeostasis. Inappropriate activation of the Hedgehog signalling pathway leads to cancers such as basal cell carcinoma and medulloblastoma, and Smoothened inhibitors are now available clinically to treat these diseases. However, resistance to these inhibitors rapidly develops thereby limiting their efficacy. The determination of Smoothened crystal structures enables structure-based discovery of new ligands with new chemotypes that will be critical to combat resistance. In this study, we docked 3.2 million available, lead-like molecules against Smoothened, looking for those with high physical complementarity to its structure; this represents the first such campaign against the class Frizzled G-protein coupled receptor family. Twenty-one high-ranking compounds were selected for experimental testing, and four, representing three different chemotypes, were identified to antagonize Smoothened with IC_50_ values better than 50 μM. A screen for analogs revealed another six molecules, with IC_50_ values in the low micromolar range. Importantly, one of the most active of the new antagonists continued to be efficacious at the D473H mutant of Smoothened, which confers clinical resistance to the antagonist vismodegib in cancer treatment.

## Introduction

Smoothened (Smo) and Frizzled (Fzd) seven transmembrane proteins form the class F or Frizzled family of G protein-coupled receptors (GPCR) [1]. Despite being conserved from fly to vertebrates, this family has low sequence identity with other GPCR classes (3–15% identity and 2–31% similarity in the transmembrane segment in human). Both Wnt and Hedgehog (Hh) ligands, signalling through Fzd and Smo respectively, play critical roles during embryonic development and adult tissue homeostasis, regulating the growth and differentiation of progenitor cell populations. Mutations or epigenetic mechanisms leading to hyperactivation of these pathways are common in human tumors [2].

Efforts to develop Hh inhibitors and Smo antagonists have been successful, as highlighted by the clinical development of vismodegib [3,4] for the treatment of cancers associated with elevated Hh pathway activity. While therapeutically effective, treatment with Smo inhibitors rapidly leads to resistance due to mutations within Smo or downstream ligand-independent pathway activation [5–7]. Notably, Smo mutations at D473 are frequently found in resistant tumours and were shown to inhibit vismodegib binding [5,8], while other known ligands, like taladegib, were reported to be unaffected or have minor drop in efficacy in the resistant mutants [9,10]. Identification of Smo inhibitors with new chemotypes or mechanisms of action may help prevent the emergence of resistance or provide secondary lines of treatment.

The recent determination of Smo crystal structures now offers the possibility to perform large structure-based screens for new antagonists [9,11,12]. In such docking campaigns, molecules are sequentially fit into a binding site, and well-fitting, high-scoring molecules are selected. Whereas these docking screens have well-known liabilities [13], they can sample a relatively large chemical space, typically between three and twelve million molecules, and can identify chemotypes unanticipated in previous screening or medicinal chemistry campaigns [14–16]. Focusing on readily available molecules—often sourced from commercial vendors—ensures that hits may be tested rapidly, reducing the cost of the false-positives made inevitable by docking approximations [17–25]. The technique has been particularly successful against GPCR structures, with hit rates of 17 to 58% (defined by the number of active molecules/number physically tested), and affinities in the 100 pM to 3 μM range, straight from the screens [17,26–35].

Here we screened 3.2 million commercially available lead-like molecules against the crystal structure of Smo, seeking those that complemented the Smo transmembrane binding site, but that were unrelated to known Smo ligands. This led to the identification of four novel antagonists in three families, and their subsequent optimization to compounds with affinities in the low micromolar range. The potential of these molecules to avoid a Smo mutation that confers resistance to vismodegib and related drugs will be considered.

## Results

### Targeting the ligand binding site within the heptahelical domain of Smoothened

The naturally occurring teratogen cyclopamine antagonizes Smo by binding in a long, narrow cavity in the heptahelical site of the protein [36,37]. This cavity broadly overlaps with that of orthosteric sites of family A GPCRs, and can accommodate at least two pharmacologically separate sites for antagonists: one at the top of the transmembrane domains and involving the extracellular loops, such as for LY2940680, and one deeper in the heptahelical bundle, such as for SANT-1 [9]. When we began this study, the only available structure was the complex with LY2940680 (PDB ID 4JKV [11]); subsequently, four other ligand structures have been published [9,11,38]. We targeted the upper 7TM site of 4JKV for docking, which also includes aspects of the second, deeper site.

### Control docking screens for enrichment of ligand vs decoys

As a positive control, we docked a library of 308 known Smo ligands, drawn from ChEMBL 12 [39], combined with 21,250 property matched decoy molecules, which had the same physical properties as the ligand set but were topologically unrelated to these 308 ligands [40]. We looked for sampling and scoring parameters that enriched the ligands over the decoys among the top-ranked molecules from this screen, using an adjusted Log(AUC) [41]; this counts the number of true ligands versus decoy molecules among the ranked molecules, weighing each log-tranche of the ranked list equally (e.g., the ratio of ligands and decoys ranking among the top 0.099% of the docking screen are weighted equally with those ranking in the next 0.1% to 0.99%, and with those in the 1% to 9.9% tranche; this serves to up-weight the early enrichment that is most relevant for docking). We found that increasing the magnitude of the local partial atomic charges of Asn219, Asp384, and Arg400, at their terminal atoms, without changing the overall charge of the residues, improved ligand enrichment; this is a technique we have used previously to up-weight the electrostatic component of the docking score relative to non-polar terms [28], hoping to improve specific recognition. The resulting adjusted Log(AUC) was 16.6%. To put this in perspective, among the top 500 docked compounds from the close to 22,000 docked, 116 were known ligands. We suspect the enrichment would have been higher still, but many of the ligands were too large to fit the particular conformation of the site represented by 4JKV.

### Prospective full library docking screen–selection of 21 compounds

We used DOCK3.6 to screen the clean lead-like subset of ZINC [42,43], then just over 3.2 million commercially available compounds, with molecular weight < 350 amu, xlogP < 3.5, and ≤ 7 rotatable bonds. Each library molecule was screened in an average of 213.3 orientations in the site, and in each orientation an average of 745.4 conformations was sampled. Overall, over 1.4 trillion molecular complexes were evaluated. Configurations were ranked according to their electrostatic (using a point charge model of the Poisson-Bolzmann equation, as implemented in QNIFFT [44,45], a version of DelPhi) [46,47] and van der Waals complementarity (using the AMBER potential [48]) to Smo, corrected for ligand desolvation (using GB/SA electrostatics as implemented in AMSOL [49,50]), and the top scoring configuration of each molecule was retained. The screen took 183 core hours on our lab cluster.

The result of the calculation was a ranked list of library molecules, from most to least complementarity to the targeted Smo ligand-binding pocket. As the differences in docking scores among the topped ranked molecules were substantially less than the expected errors of the calculation, we winnowed to a final candidate list for testing by visual inspection, as is commonly done in both high-throughput and virtual screening [26,51]. We inspected the top 0.2% of the docking-ranked library, seeking compounds predicted to form hydrogen bonds with at least two of the residues known to be important for binding the known antagonists (Asn219, Asp473, Arg400, Lys394, Glu518 and Asp384). To bias toward novel scaffolds, we selected not only the compounds that overlapped with the LY2940680 binding site, but also some that bound higher in the site and only partially overlapped with this ligand in the structure. We deprioritized those molecules that were conformationally strained (e.g. in cases with polar atoms, often protons or hydroxyl groups, that were too closely juxtaposed), something not always well captured by the docking scoring function and described in the past [23,26,52], and selected for molecules with diverse chemotypes. Ultimately, 21 compounds were selected for experimental testing (S1 Table). All showed specific and satisfactory electrostatic interactions, reasonable poses, and represented different chemotypes compared to known ligands and typically to each other.

**Fig 1:**
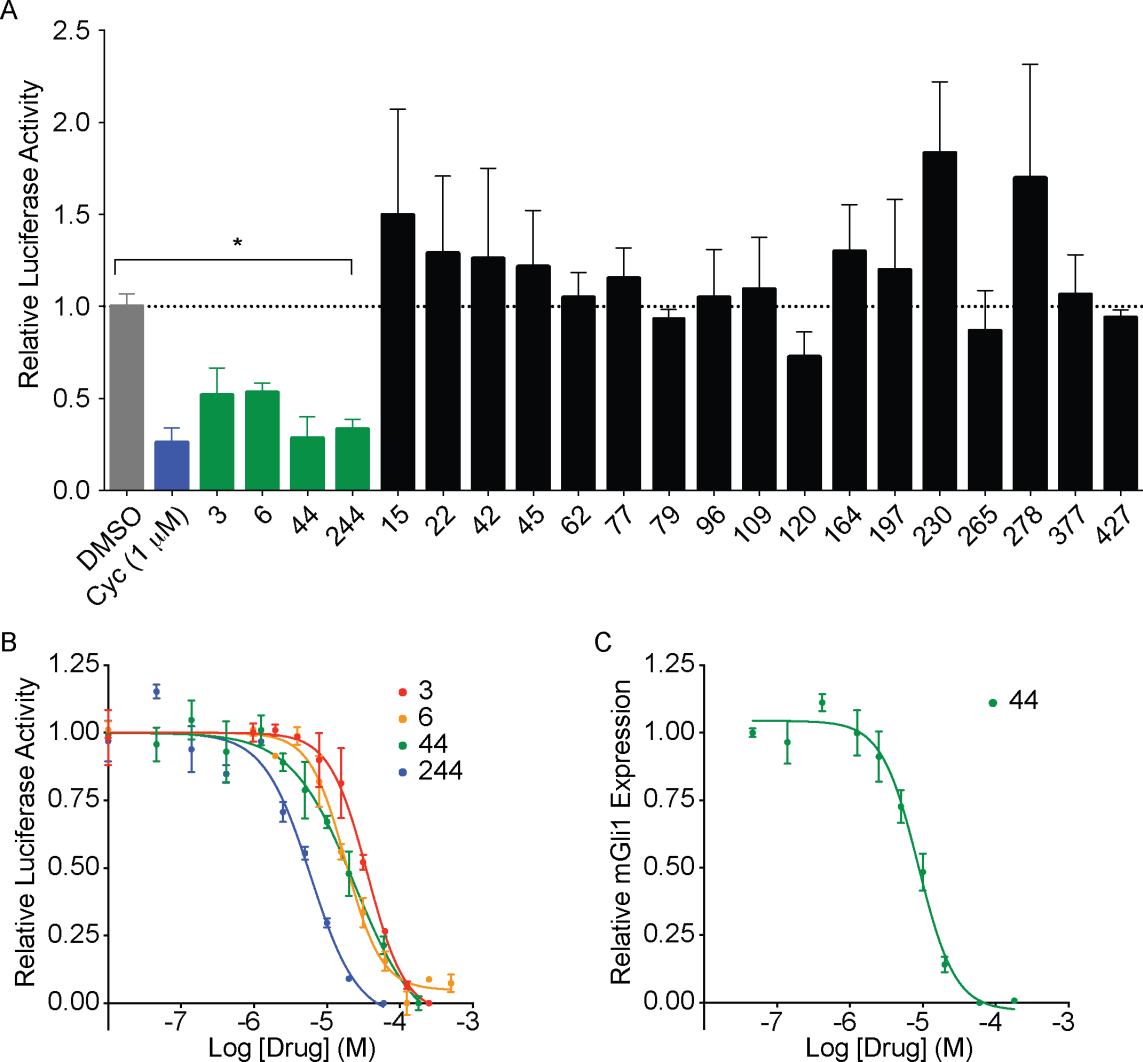
Identification of four novel Smoothened antagonists. (A) First screen of 21 compounds at 30 μM using the 8xGli-Luciferase reporter in *Ptch1*^-/-^ MEFs. Four compounds showed significant (>50%) inhibition of the reporter compared to DMSO, n = 3, p<0.05. cyc: cyclopamine (1 μM). (B) Dose-response analysis of initial hits using 8xGli-Luciferase reporter in *Ptch1*^-/-^ MEFs. (C) Dose-response analysis of compound 44 by qPCR of *Gli1* expression in *Ptch1*^-/-^ MEFs. n = 3, combined experiments, error bars: standard deviation.

**Fig 2:**
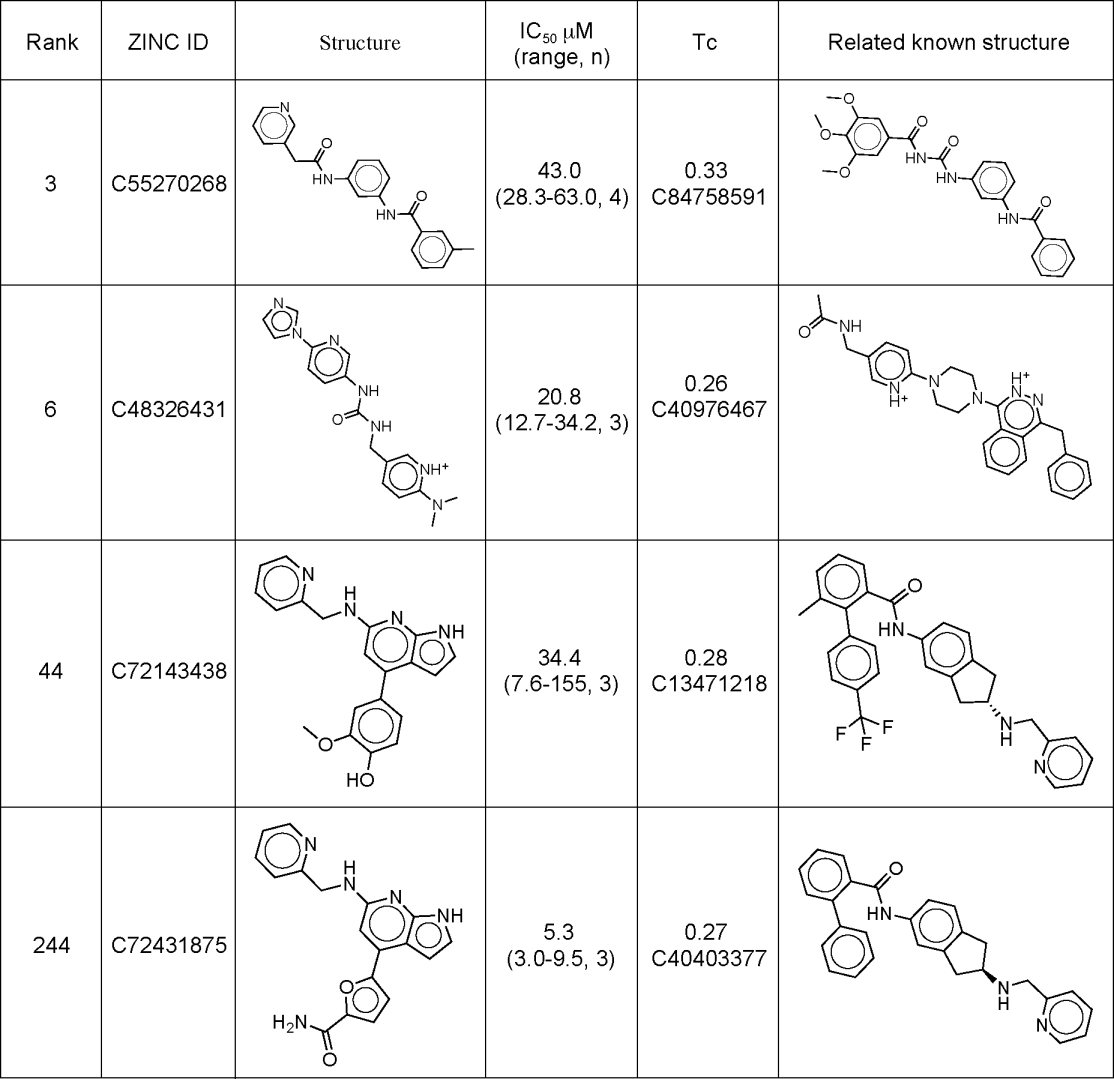
Structures of initial hits. The table includes their IC50 in the Gli-Luciferase assay in *Ptch1*^-/-^ MEFs, and their Tanimoto coefficient (Tc) score compared to the closest known Smo ligand.

Antagonist candidates were tested using *Ptch1*^-/-^ reporter mouse embryonic fibroblasts (MEFs). In *Ptch1*^-/-^ cells, the Hh pathway is constitutively activated as a result of deletion of Patched-1, the Hedgehog receptor and a functional inhibitor of Smo. The reporter cells were engineered to express the 8XGli-Luciferase reporter that faithfully monitors levels of Gli-mediated transcriptional activity as a readout of Hedgehog signalling. Firefly Luciferase is therefore constitutively expressed in these cells and its expression is inhibited by Smo antagonists such as cyclopamine [36]. In the initial test for activity, we screened the 21 docking hits at a dose of 30 μM. From these, four molecules **3**, **6**, **44** and **244** (number indicates rank from the docking screen) exhibited greater than 50% inhibition of the reporter (Figs 1A and 2). These compounds repressed the reporter in a dose dependent manner, with compounds **44** and **244** displaying IC_50_ of 34.4 μM and 5.3 μM respectively (Fig 1B). Their ability to repress the pathway was further confirmed by quantifying transcript levels of *Gli1*, a target gene of the Hedgehog pathway, using qPCR (Fig 1C and S1 Table).

### Secondary screen identifies analogs

**Fig 3:**
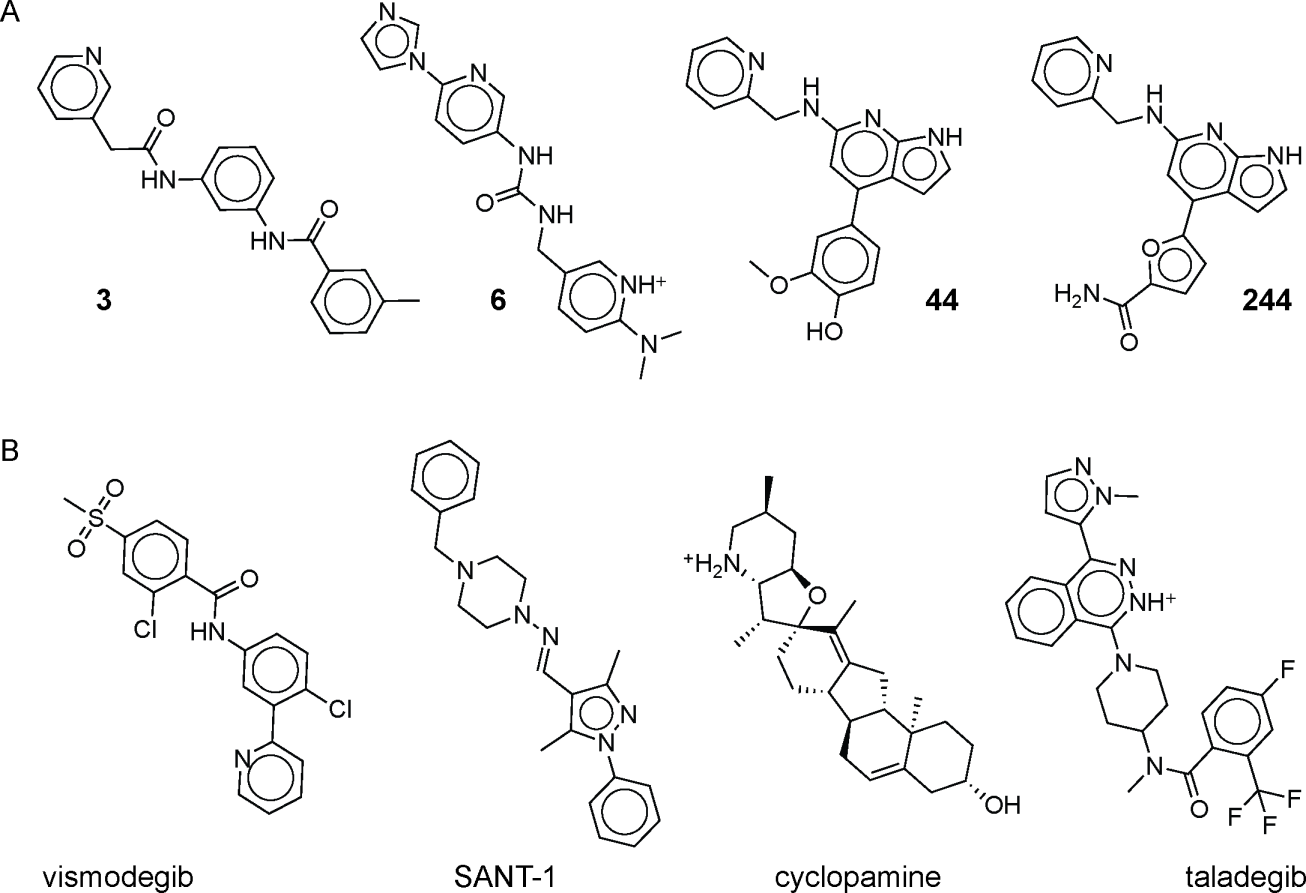
Smo antagonist structures. (A) 2D structures of the four novel antagonists from the initial screen (left to right: compounds **3**, **6**, **44** and **244**). (B) 2D structures of known antagonists (left to right: vismodegib, SANT-1, cyclopamine, and taladegib (LY2940680)).

**Fig 4:**
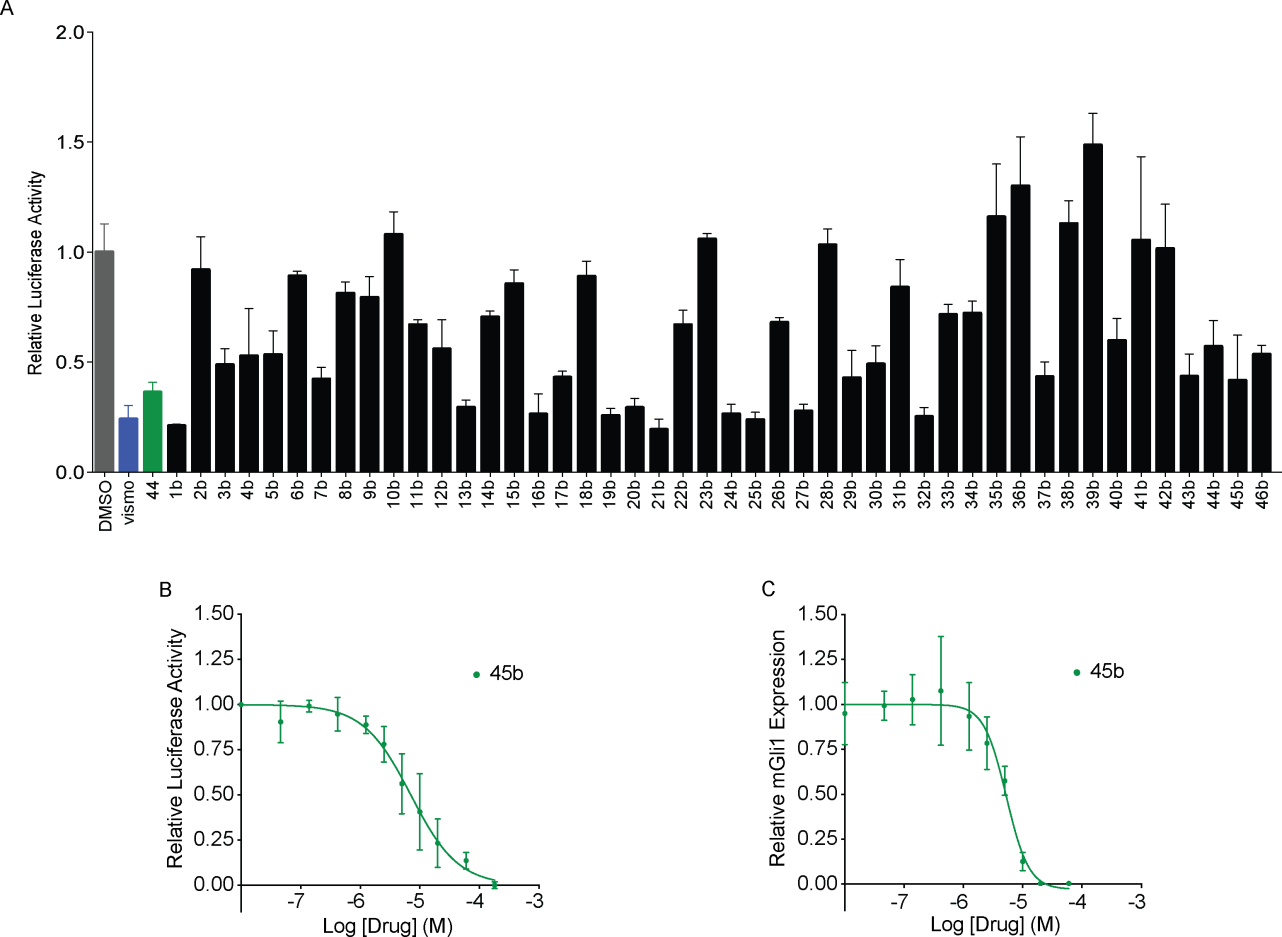
Screening of analogs and dose-response analysis of best hit. (A) Second screen at 30 μM using the 8xGli-Luciferase reporter in *Ptch1*^*-/-*^ MEFs. vismo: vismodegib at 100 nM. (B) Dose-response analysis of compound **45b** using the 8xGli-Luciferase reporter in *Ptch1*^*-/-*^ MEFs. n = 3 (C) Dose-response analysis of compound **45b** by qPCR of *Gli1* expression in *Ptch1*^*-/-*^ MEFs. n = 3, combined experiments, error bars: standard deviation.

**Fig 5:**
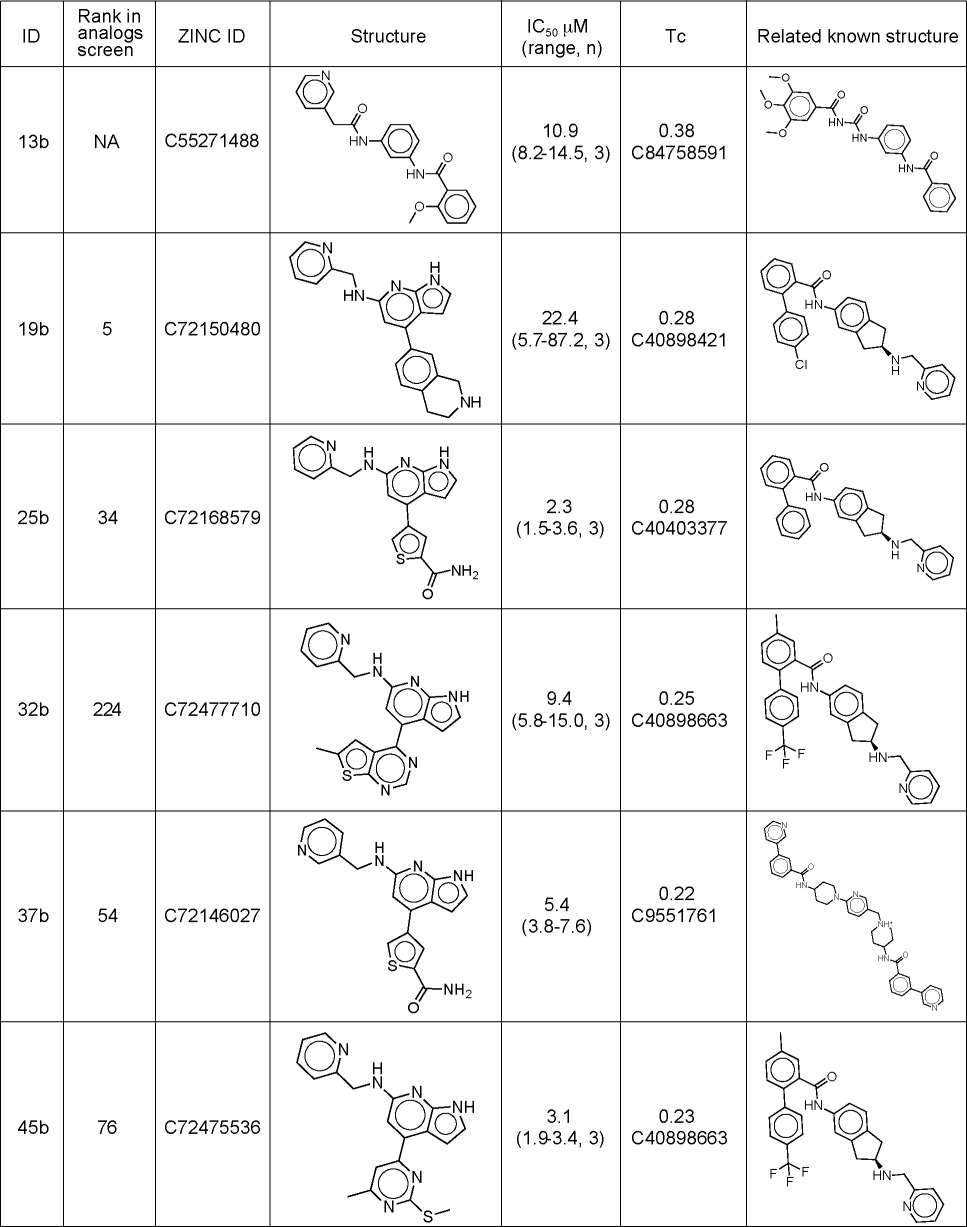
Structures of the antagonists discovered in the analogs screen. The table includes their IC_50_ in the Gli-Luciferase assay in *Ptch1*^*-/-*^ MEFs, and their Tc score compared to the closest known Smo ligand.

In an effort to improve affinity, we searched for commercially available analogs of the first four hits. Any compound in the ZINC database [43,53] within an ECFP4-based Tanimoto coefficient (Tc) of 0.7 to any of the four hits was considered (representing high topological similarity) [54]. Many such compounds were available for compounds **44** and **244**, and we selected 231 that either fit within the similarity cut-off for compound **244** or bore the chemical scaffold common to both **44** and **244** (Fig 3A). Only one analog was available for compound **3**, and none were available for compound **6**. Because most were larger than the initial lead-like molecules docked, they had not been sampled in the original docking screen. Thus, the entire set of analogs was docked against the Smo structure. Many scored well, and 190 would have ranked among the top 0.5% of compounds from the original screen. Of these, 46 were purchased and tested (compounds **1b**-**46b**). Thirty of these antagonized the reporter at a single dose (Fig 4A, S2 Table), and several had IC_50_ values in the low micromolar range, including compounds **13b, 25b**, **32b** and **45b** at 10.9 μM, 2.3 μM, 9.4 μM and 3.1 μM respectively as determined using the Gli-Luciferase assay and/or by measurement of *Gli1* levels using qPCR (Figs 4B, 4C and 5).

All the antagonists with low micromolar IC_50_ were counter-screened for colloidal aggregation, a common mechanism of artefactual activity in early ligand discovery [55–57]. Dynamic Light Scattering (DLS), centrifugation of putative colloidal aggregates in media, and counter-screening assays against unrelated enzymes were used to confirm that compounds **3**, **6**, **44**, **244**, **25b**, **32b**, **37b** and **45b** are well-behaved antagonists (S1 Fig). Four compounds were found to be aggregators in one or more assays (S3 Table). Intriguingly, the same behaviour was observed for the anti-fungal drug itraconazole, which has been promoted into Phase II clinical trials [58] after it was discovered to act as a Smo antagonist in a drug repurposing screen [59,60]. Itraconazole was previously shown to be a potent aggregator, active against several GPCRs in the 200 nM to 2 μM range via this artefactual mechanism [57]. Consistent with this behaviour, we found that itraconazole formed colloidal particles of radius 180 nm, with a critical aggregation concentration just below 1 μM (S2 Fig), and that its observed antagonism of Smoothened could be disrupted by prior-centrifugation, a harbinger of this mechanism (S3 Fig). These observations highlight the importance of counter-screening for this artefactual mechanism of action when evaluating new Smoothened antagonists.

**Fig 6:**
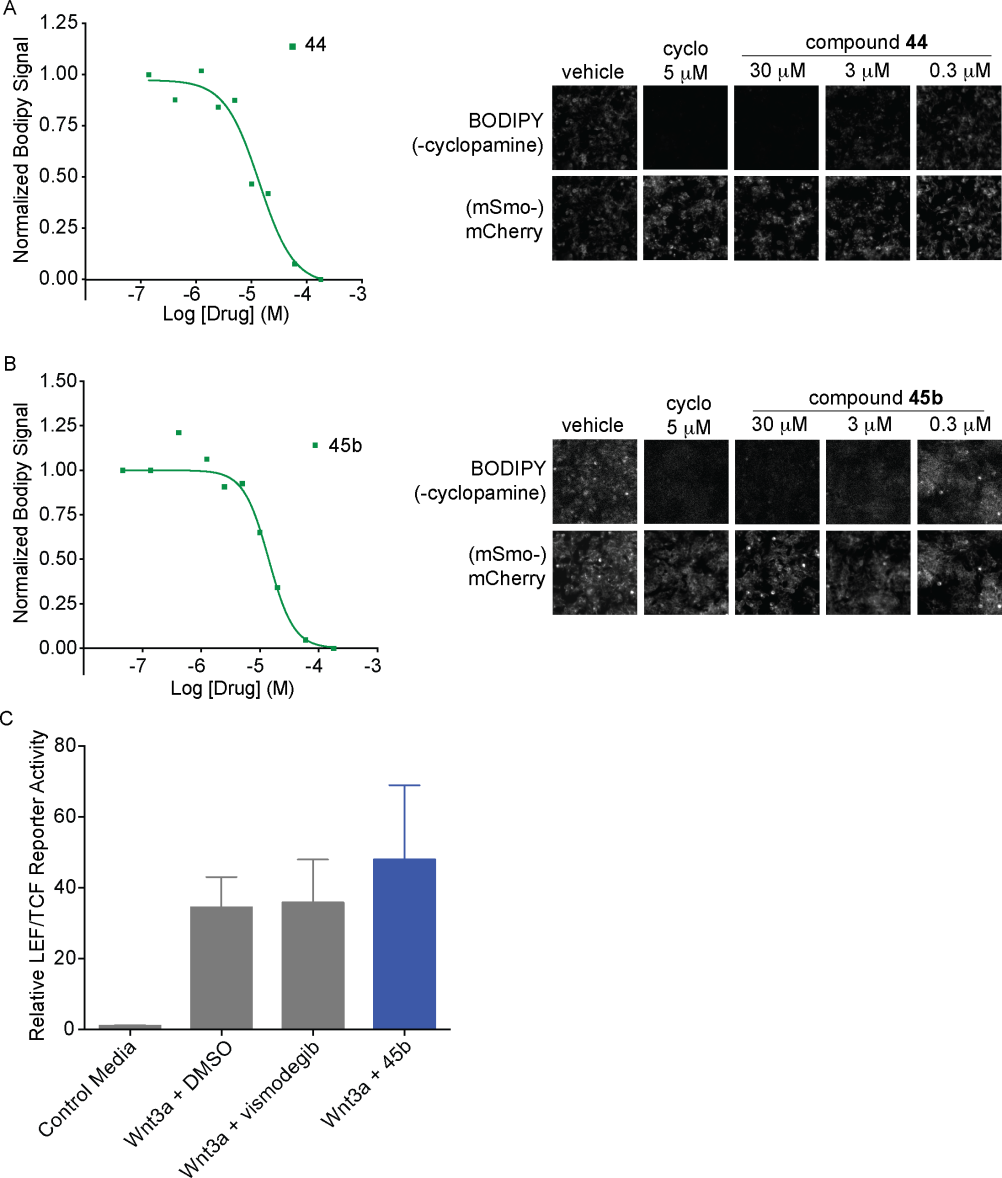
New Smo antagonists compete for BODIPY-cyclopamine binding. (A) and (B) Dose-response analysis of BODIPY-cyclopamine displacement in HEK293 cells overexpressing mSmo-mCherry for compound **44** (A) and compound **45b** (B). Left: average cell fluorescence was measured by flow cytometry and plotted against antagonist concentration. Representative curve are shown. Right: representative images of BODIPY-cyclopamine displacement by antagonists in HEK293 cells overexpressing mSmo-mCherry. Cells were incubated with 5 nM BODIPY-cyclopamine and compounds **44** or **45b** for 2 hours. (C) Specificity of **45b** towards Smo is demonstrated by the lack of inhibition of the TOPFLASH Wnt-ß-catenin reporter by compound **45b** (10 μM) and vismodegib (100 nM). n = 3, combined experiments, error bars: standard deviation.

Displacement of a BODIPY-derivative of the canonical Smoothened ligand cyclopamine has been previously used to determine the binding affinity of Smo modulators [61]. Using a stable line enabling the inducible expression of Smo-mCherry, we tested whether well-behaved, non-aggregating antagonists can specifically displace BODIPY-cyclopamine bound to Smoothened using flow cytometry. Compounds **44** and **45b** had IC_50_ values of 15.6 μM and 12.7 μM in this ligand-displacement assay (Fig 6A and 6B, S1 and S2 Tables), suggesting that the binding site for these compounds overlaps with the one occupied by BODIPY-cyclopamine. To further validate the specificity and rule out off-target activity for this new Smo antagonists chemotype, we investigated the activity of compound **45b**, the most potent antagonist discovered in this study, against Frizzled receptors. Vertebrate genomes encode ten Frizzled proteins, which function as receptors for Wnt growth factors, and with Smo they constitute the class F family of GCPRs. We used HEK293T TopFlash cells, expressing a luciferase reporter under the control of a ß–catenin-responsive LEF/TCF promoter. Wnt3a-conditioned media was used to activate the pathway and potential activity of compound **45b** was measured after 24 hours of co-treatment (Fig 6C). Compound **45b** had no detectable activity in this assay suggesting that it does not interact with Frizzled receptors. We conclude that compound **45b** and the other analogs represent a new chemotype for Smo antagonists.

### The new antagonist exhibits efficacy at the chemoresistant Smo-D473H mutant

**Fig 7:**
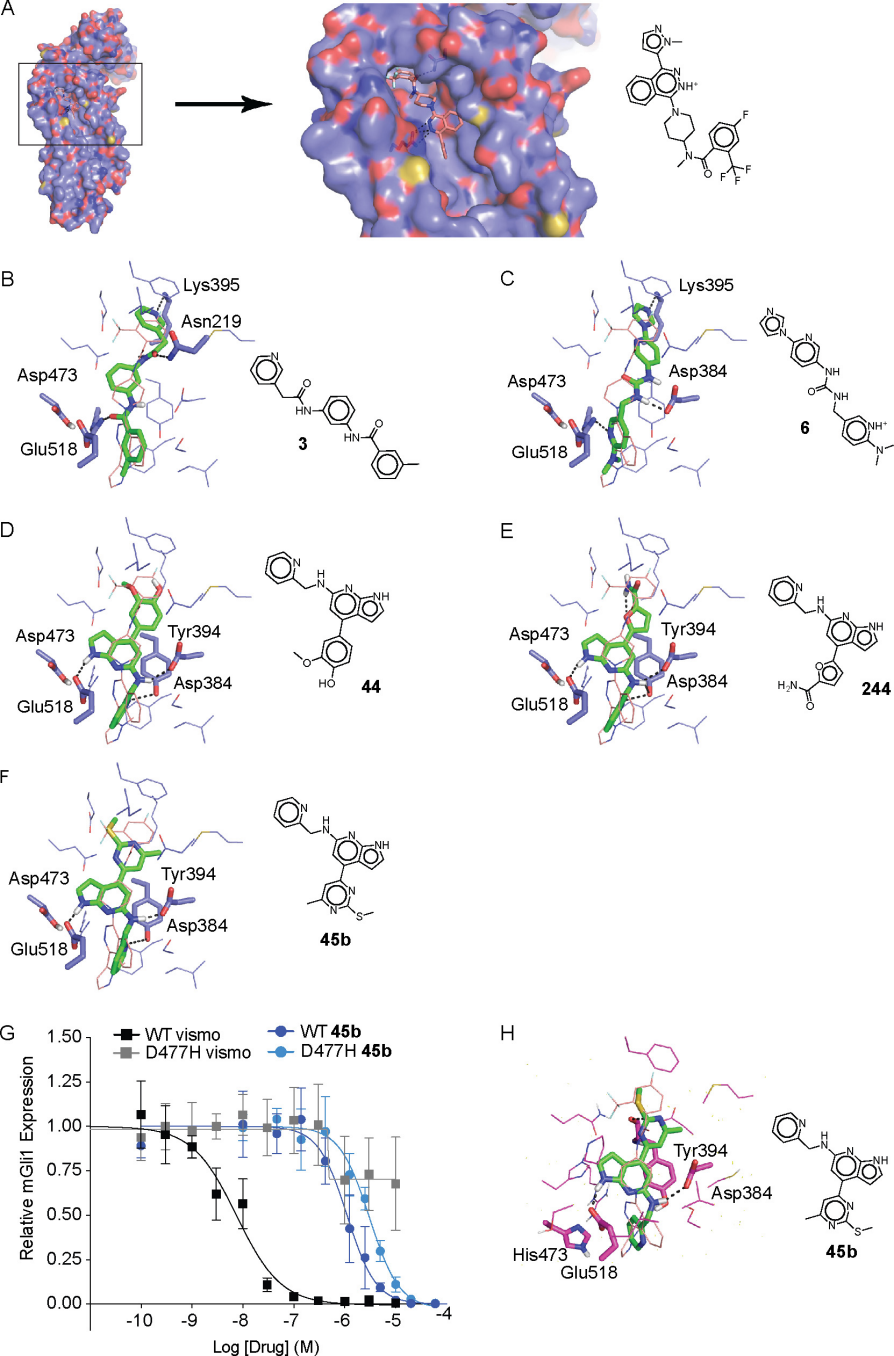
Binding poses and inhibition of vismodegib-resistant Smo by 45b. (A) Side view of the complex of Smo structure (represented as purple surface, TM6 was cut) with LY2940680 in the orthosteric site (represented as light pink sticks). (B)-(F) Predicted binding modes against the Smo WT of compounds **3**, **6**, **44**, **244** and **45b**, respectively. LY2940680 is represented as light pink wires, the compounds as green sticks, hydrogen bonds as black dashed lines and important residues as sticks. (G) Compound **45b** inhibits Smo wt and mouse D477H mutant (equivalent to human D473H) in a dose-dependent manner. n = 3, combined experiments, error bars: standard deviation. C3H10T1/2 cells were transduced with retrovirus for the overexpression of Smo wt or Smo D477H. (H) Predicted binding modes of compound **45b** against a model of D473H Smo.

Compounds **3**, **6**, **44**, **244**, **25b**, **32b**, **37b** and **45b** were all docked within the heptahelical bundle of Smo, where other Smo ligands like taladegib (LY2940680) also bind (Fig 7). However, these new antagonists are broadly unrelated to previously known Smo antagonists (Fig 3B), and none has an ECFP4-based Tanimoto coefficient (Tc) [62,63] greater than 0.38 when compared to any Smo antagonist in the ChEMBL19 database [39,64] (Figs 2 and 5). This is particularly true of compounds like **45b**, which bears a Tc of only 0.23 to the nearest known Smo antagonist, and a Tc of only 0.12 to taladegib, indicating that these molecules not only represent scaffold hops [65] but have little more similarity than would be expected among randomly selected lead-like or drug-like molecules (Fig 5) [66]. Structurally, the docking hits are also dissimilar to the lead. In the crystallographic complex with Smo, taladegib hydrogen bonds with Arg400 and Asn219, and makes hydrophobic interactions with residues from ECL3, including Gln477, Trp480, Glu481 and Phe484, which stacks with the phenyl ring of the ligand (Fig 7A). Compound **45b**, consistent with the new scaffold it represents, makes interactions completely different from taladegib with hydrogen bonds with Glu518, Asp384 and Tyr394, and stacking with Tyr 394 (Fig 7E). Encouraged by the unique docked pose of **45b**, which doesn’t interact with Asp473, we tested compound **45b** against the D473H mutant of Smo, which was reported to mediate the clinical resistance to vismodegib [5]. This mutation reduces vismodegib binding to Smo 100 folds, whereas binding of compound **45b** is only 2.7-fold relative to WT (1.1 μM to 3.1 μM, Fig 7G). Docking of compound **45b** to a model of Smo-D473H, after a minimization with the AMBER program suggested a docking pose with hydrogen bonds with Glu518 and Asp384 (Fig 7H), while His473 slightly moves and does not interfere with the binding of **45b**. Whereas the resilience of **45b** to this mutant was not a feature that was selected for at the time of docking, and is in this sense fortuitous, it highlights the uniqueness of its chemotype. Such novelty was revealed in the docking results, and in general the ability to discover novel scaffolds and chemotypes is an advantage one can reasonably hope for in a docking screen.

## Discussion

Three results merit emphasis. First, a structure-based approach discovered several novel scaffolds unrelated to previously described Smo inhibitors. Second, these new antagonists made interactions distinct from previous ligands within the canonical Smo intra-helical binding sites and one of the most potent, compound **45b**, was little affected by the D473H mutation in Smo previously shown to limit vismodegib clinical efficacy. Finally, we confirmed the mechanism of binding of these compounds, investigating them not only by functional assay but by fluorescent-ligand displacement and controlling for colloidal aggregation. This artefactual mechanism indeed affected four of the 14 antagonists discovered herein, as well as the highly studied Smo antagonist itraconazole, which may therefore also behave as a colloidal aggregator against this target.

Molecular docking screens have proven effective for GPCRs, partly because of the relatively high bias among commercially-available compounds towards relevant chemotypes [23,27] but also because of the ideal ligand binding cavity within the trans-membrane helical domains. This is a feature that Smo shares: its site is largely closed off from bulk solvent, and although larger than the orthosteric sites of aminergic GPCRs like the ß2 receptor, it is substantially smaller than that of peptide GPCRs like the μ-opioid receptor. Both burial from solvent and a well-formed site contribute to good ligand complementarity, which is important for docking success. As with other GPCR docking campaigns, the initial hit rate against Smo was higher than we typically observe against soluble proteins, though at 19% (4 active out of 21 tested) it is at the lower end of the range we have observed against other GPCRs (ranging from 17% to 58%). The potency of the hits, which were in the 2 to 25 μM range, were one to two logs weaker than observed in most other GPCR campaigns. Several factors may have contributed to these results. First, there is a substantial bias of commercially available libraries towards well-investigated GPCR-like chemotypes, such as ligands of the β2-adrenergic receptor (ADRB2), serotonin receptor 2A (HTR2A) or dopamine receptor D2 (DRD2) (S4 Table). Illustrating this, there are 26,137 purchasable analogs for the known ligands of D2 receptor (with affinities below 1 μM) and only 2,835 purchasable molecules that resemble known Smo ligands. Second, testing more compounds with different scaffolds in the initial screening might have increased the hit rate. Third, our main goal here was to find novel chemotypes, while in earlier campaigns many of the hits resembled known ligands and recapitulated canonical interactions [7,67]. Insisting on novelty likely reduces the probability of finding higher affinity hits that exploit already developed chemotypes, but it has the advantage of finding antagonists with new properties.

The new antagonists, though docked in the canonical LY2940680/cyclopamine site, were predicted to make interactions that differ from these compounds, as defined in the Smo crystal structures. Crucially, the most potent antagonist, **45b**, interacted with Glu518 and Asp384 in its docked WT complex. Docking and minimization in the modeled D473H mutant structure resulted in a very similar pose. This predicted behaviour is consistent with the experimental results showing that compound **45b** is resilient to the resistance-conferring mutation D473H. Although we did not predict this from the start, or even select for it, we did aim for novelty and selected compounds forming interactions with other residues than the crystal structure.

As in so many other early discovery campaigns, some of our initial hits turned out to be colloidal aggregators—this is an artifact to which Smo is clearly prone. Four out of the 14 analogs that we discovered were aggregators in one or more assays. This emphasizes the importance of controlling for mechanism in early discovery campaigns against this and related targets. This same aggregation mechanism may also affect a heavily studied Smo antagonist, indeed one advanced into the clinic, the popular repurposing drug itraconazole.

Certain caveats bare airing. Despite the investigation of almost 50 analogs, activity of the new antagonists never breached 1 μM. Whereas there is room to optimize these molecules, several of which have ligand efficiencies > 0.3 (ΔG_bind_/heavy-atom-count > 0.3), their affinity remains well outside of the range desired for leads or probes. Although the resilience of **45b** to the D473H mutant is consistent with its docked geometry, other binding modes are possible. Finally, whereas colloidal aggregation is a concern for GPCRs in general [57] and for Smo in particular, its role in the activity of itraconazole has not been fully defined. Undoubtedly itraconazole is a strong aggregator, and it aggregates in the range of its Smo activity, but whether its activity on Smo may be laid exclusively at the door of colloidal aggregation remains uncertain.

These caveats should not obscure the major observations of this study. A structure-based screen found ten new antagonists in three new scaffolds for Smo. One of the most potent, compound **45b**, retained its activity against the D473H mutant of Smo that confer clinical resistance to vismodegib. Our study therefore leveraged the strength of structure-based docking to identify ligands with new chemotypes for Smo, a class F GPCR. As more structures become available, this approach may also enable the identification of Frizzled ligands, for which no small molecule modulators are currently available and highly sought considering their implications in human diseases such has cancer.

## Methods

### Docking against Smo WT

A set of Smo 308 ligands were extracted from CHEMBL 12 [39] with a cut-off of 10 μM affinity. About 21,000 decoys property-matched to these ligands were calculated using the decoy generation tools at the DUD-E site (http://dude.docking.org). We used DOCK 3.6 [41] to screen the “lead now” subset of the ZINC database (http://zinc.docking.org) with properties of xlogP ≤3.5, molecular weight ≤ 350 Dalton and ≥ 250 and rotatable bonds 7 [43,53] against the x-ray crystal structure of the human Smo bound to an antagonist LY2940680 (PDB ID 4JKV) [11]. About 3.2 million molecules were screened against the Smo orthosteric site. Complementarity of each ligand pose was scored as the sum of the receptor-ligand electrostatic (using ligand probe charges in an electrostatic potential calculated by QNIFFT [44,45], a version of DelPhi [46,47]) and van der Waals interaction energy (using the AMBER potential [48]) and corrected for ligand desolvation. Partial charges from the united-atom AMBER force field were used for all receptor atoms except for Asn219, Asp384 and Arg400 for which the dipole moment was increased as previously described [28] to boost electrostatic scores for poses in polar contact with these important residues. Forty-five matching spheres were used. The degree of ligand sampling is determined by the values of the bin size, bin size overlap and distance tolerance, set at 0.3Å, 0.1Å and 1.2Å, respectively, for both the matching spheres and the docked molecules. Ligand internal degrees of freedom were pre-calculated using Openeye’s Omega program [68]. Ligand charges and initial solvation energies were calculated using AMSOL (http://comp.chem.umn.edu/amsol/) [49,50].

### Tanimoto coefficient (Tc) calculation

Subsequently, an updated dataset of 452 ligands, this time extracted from the by now more recent CHEMBL19 [39,64] was used. Using the GenerateMD program (version 5.10.3) in the Chemaxon package we calculated the EFCP4 fingerprints which were used to calculate the Tc [63] between our hits and all of the 452 ligands.

### Modeling, docking and minimization with AMBER against D473H mutant

PyMol software was used to build the model of mutant D473H. Using DOCK 3.6 [41] we docked compound **45b** towards this mutant and using the AMBER molecular mechanics program [69,70] we minimized the complex of the mutant model with compound **45b**. The starting structures were taken from the docked pose. The structures were subjected to 10,000 steps of conjugate gradient minimization.

### Luciferase assay

*Ptch1*^*-/-*^ MEFs stably expressing the Gli-Luciferase reporter and constitutive Renilla Luciferase were used. The Gli-Luciferase reporter is a Firefly Luciferase reporter driven by 8xGli consensus binding site in its promoter, cloned in a lentiviral plasmid carrying a puromycin resistance for selection. For the assay, 5x10^4^ cells/well were plated in 48-well plates. The next day, the confluent cells were serum-starved with plain DMEM for 24 h. Drugs and compounds were added to the indicated final concentration and incubated for 24 h, each condition in duplicate. For the assay, Promega Dual Glow reagents were used. Media was removed and cells were lysed in 50 μL Passive Lysis Buffer for 10 min. 10 μL of lysate was assayed in black plates with 10 μL of each substrate, in duplicate. Luminescence was measured with an EnVision 2100 (Perkin Elmer). Firefly Luciferase luminescence was divided by the Renilla Luciferase luminescence, then normalized to vehicle condition to obtain the fold change in reporter activity.

### Aggregation counter-screens

The assays were performed as always with the addition of a centrifugation step. The antagonists were diluted in media to their final concentration. Half the solution was used as standard treatment: the media in wells was replaced with the media containing the drugs. The other half of the solution was then centrifuged for 20 min at 21,000*g*. The supernatant was used to replace the media in the wells.

### Top-Flash assay

The assay was carried out in HEK293T, as described [71].

### RNA isolation, cDNA synthesis and qPCR analysis

*Ptch1*^*-/-*^ MEFs or C3H10T1/2 SMO cells (overexpression of mouse Smo wt or mouse Smo D477H using a retroviral vector) were plated at a density of 2x10^5^ cells/well in 12-well plates. The next day, the confluent cells were serum-starved with plain DMEM for 24 h before drugs and compounds were added to the indicated final concentration. RNA was extracted using Trizol (Life Technologies, 15596–018) after 24 h. 1 μg of RNA was DNase-treated (Life Technologies, 18047–019) before being reverse transcribed into cDNA (High-Capacity Reverse Transcription kit, Life Technologies, 4368813). Real-time quantitative PCR reactions were performed on an ABI 7900HT in 384-well plates containing 20 ng cDNA, using Power SYBR Green PCR Master Mix (Life Technologies, 4367660). Relative *Gli1* mRNA levels were calculated using the comparative Ct method, normalized to *Gapdh* mRNA. Primers used were validated as previously described [72].

### BODIPY-Cyclopamine binding assay, FACS and microscopy

A 293 stable cell line expressing tetracycline-inducible mouse Smoothened with mCherry fused to its C-terminus was used for these experiments. Cells were grown to confluence in the presence of 1 μg/mL tetracycline for 24 h. Cells were then incubated with 10 nM BODIPY-Cyclopamine and compounds for 2 h at 37°C. For FACS, cells were first trypsinized, fixed with 4% paraformaldehyde for 20 min, washed with TBS + 0.1% Triton X-100 and then sorted (FACS). BODIPY fluorescence was measured on the FACS Fortessa and FACS data was analyzed with FlowJo software. BODIPY fluorescence in control HEK293 was used to set the background threshold. Mean fluorescence was plotted against the compound concentration to calculate its IC_50_. For microscopy, fixed cells were imaged. Live cells were washed with PBS before fixing.

### Dynamic Light Scattering (DLS)

Concentrated DMSO stocks of itraconazole and vismodegib were diluted with filtered DMEM, with a final concentration of 1% DMSO. Compounds **13b**, **19b**, **20b**, **25b**, **27b**, **32b**, **37b**, **40b** and **45b** were diluted with both filtered DMEM and KPi, with a final concentration of 1% DMSO. Measurements were made using a DynaPro Plate Reader II system (Wyatt Technology) with a 60 mW laser at ~830 nm in either 96-well or 384-well plates; this particular instrument had been modified by Wyatt Technology to have a larger laser beam width that is appropriate for detecting large colloidal particles [73, 74].

### CAC determination

Normalized scattering intensities (counts/seconds, cnt/s) were plotted against decreasing concentrations of itraconazole. Data for colloidal and non-colloidal states were linearly regressed and non-linearly regressed, respectively. The intersection point between them was determined to be the critical aggregation concentration. Concentrations are represented as the mean and the standard deviation of three repetitions.

### Enzyme inhibition assays

Inhibition of AmpC ß-lactamase and MDH in counter-screening assays were measured as described [73–76]. The final concentration of DMSO was 1% for all samples. Values reported are the average of duplicate samples run in two independent experiments. Both DMEM and KPi were used as buffer.

## Supporting Information

S1 Fig Centrifugation counter screen Gli-luciferase reporter activity in *Ptch1*^-/-^ MEFs. Centrifugation had no significant effect on the activity of the antagonists tested. Errors bars: standard deviation, combined replicates, n = 3.
(TIF)

S2 Fig **Particle formation by itraconazole as measured by dynamic light scattering (DLS)** (A) 1 μM itraconazole forms strongly scattering particles dominated by those at 180 nm radius by DLS. (B) The strong DLS decay curve 1 μM itraconazole (red) is eliminated by centrifugation in a benchtop microfuge. vismodegib (green) does not form particles by DLS at 1 μM. (C) itraconazole particles transit through a critical aggregation concentration (CAC) of 0.9 ± 0.2 μM, moving from a soluble to a particulate form over a small concentration interval.
(TIF)

S3 Fig **Itraconazole inhibits Smo via an aggregation-based mechanism** Gli-luciferase reporter activity in *Ptch1*^-/-^ MEFs (left), qPCR of *Gli1* transcript in *Ptch1*^-/-^ MEFs (middle), and direct displacement of bodipy-cyclopamine (right)—(A)-(C) Effect of centrifugation on vismodegib: vismodegib antagonism of Smo is unaffected by a 20 min centrifugation of the antagonist (red) compared to control (black). (D)-(F) Effect of centrifugation on itraconazole: itraconazole activity is largely or entirely eliminated by a 20 min centrifugation of the antagonist (red) compared to control (black). Error bars: standard deviation, combined replicates n = 3.
(TIF)

S1 Table Results of first screen. (PDF)

S2 Table Results of analog screen. (PDF)

S3 Table Compounds positive in aggregation counter screen. (PDF)

S4 Table Library bias. (PDF)
