# Supplementary figures and images for: Identification of Novel Smoothened Ligands Using Structure-Based Docking
Source: PLoS One. 2016 Aug 4;11(8):e0160365. doi: 10.1371/journal.pone.0160365 (PMC4973902; doi:10.1371/journal.pone.0160365)

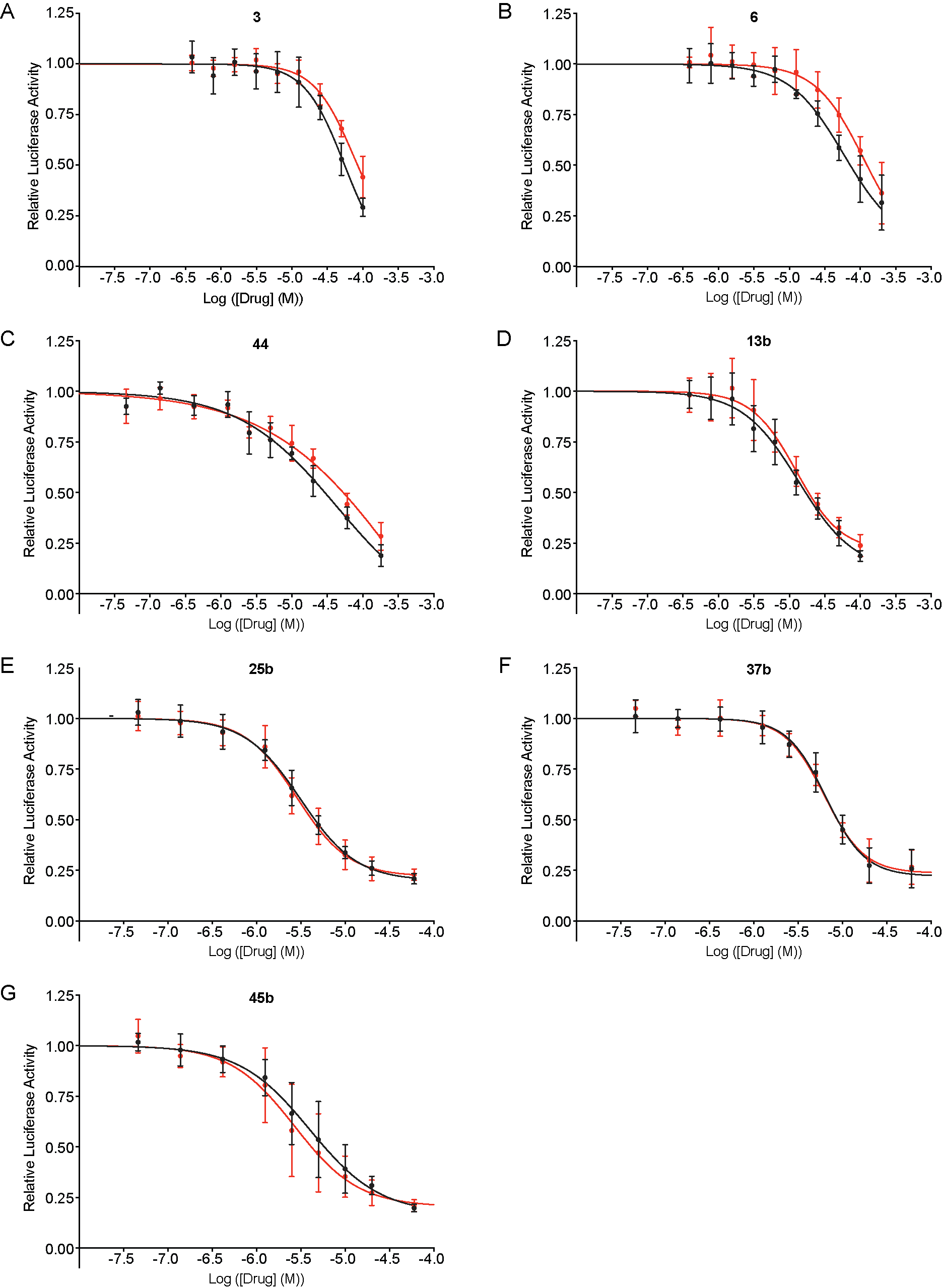

Supplement: S1 Fig — Centrifugation had no significant effect on the activity of the antagonists tested. Errors bars: standard deviation, combined replicates, n = 3. (TIF) [file pone.0160365.s001.tif]

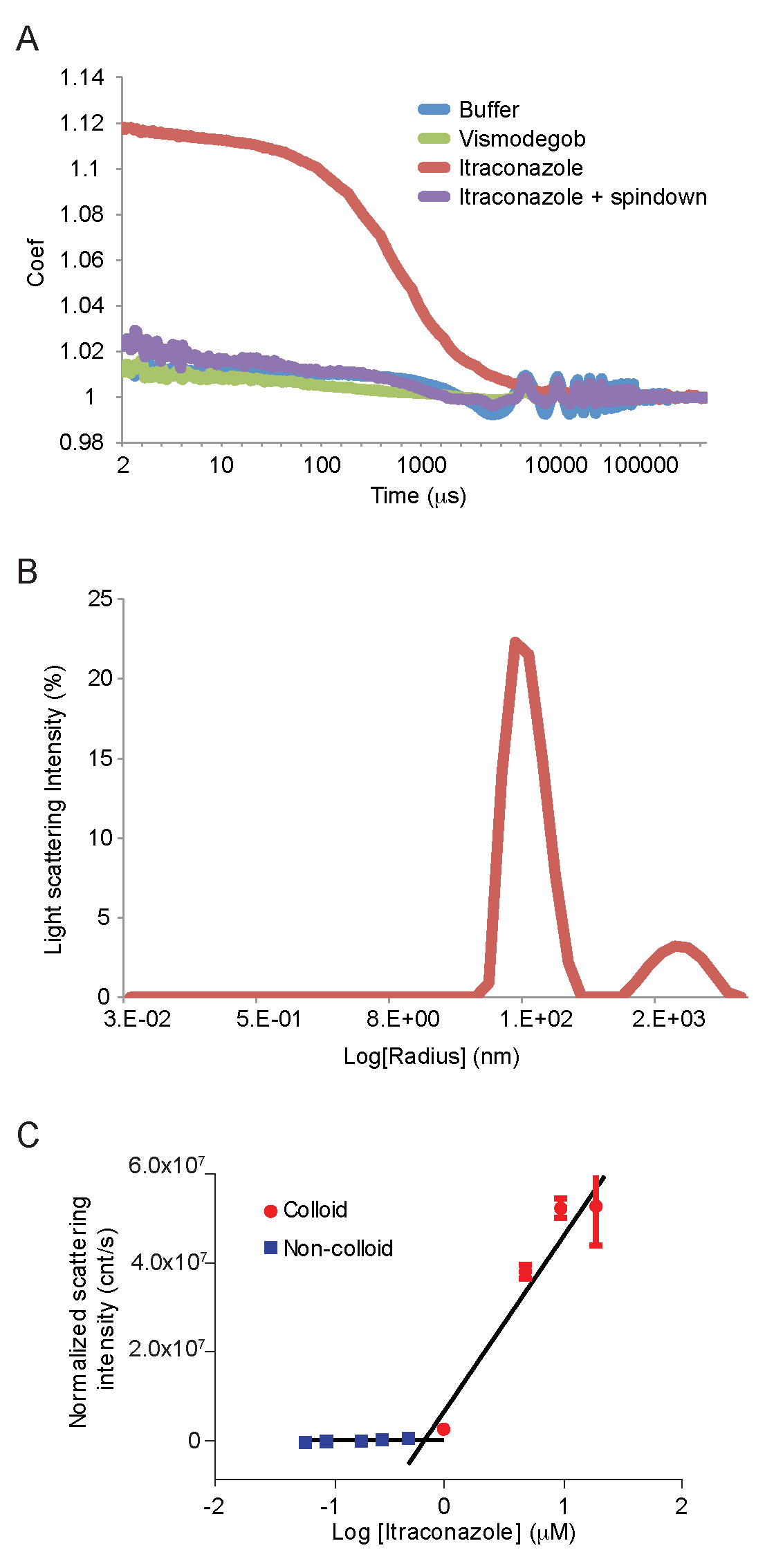

Supplement: S2 Fig — Particle formation by itraconazole as measured by dynamic light scattering (DLS) (A) 1 μM itraconazole forms strongly scattering particles dominated by those at 180 nm radius by DLS. (B) The strong DLS decay curve 1 μM itraconazole (red) is eliminated by centrifugation in a benchtop microfuge. vismodegib (green) does not form particles by DLS at 1 μM. (C) itraconazole particles transit through a critical aggregation concentration (CAC) of 0.9 ± 0.2 μM, moving from a soluble to a particulate form over a small concentration interval. (TIF) [file pone.0160365.s002.tif]

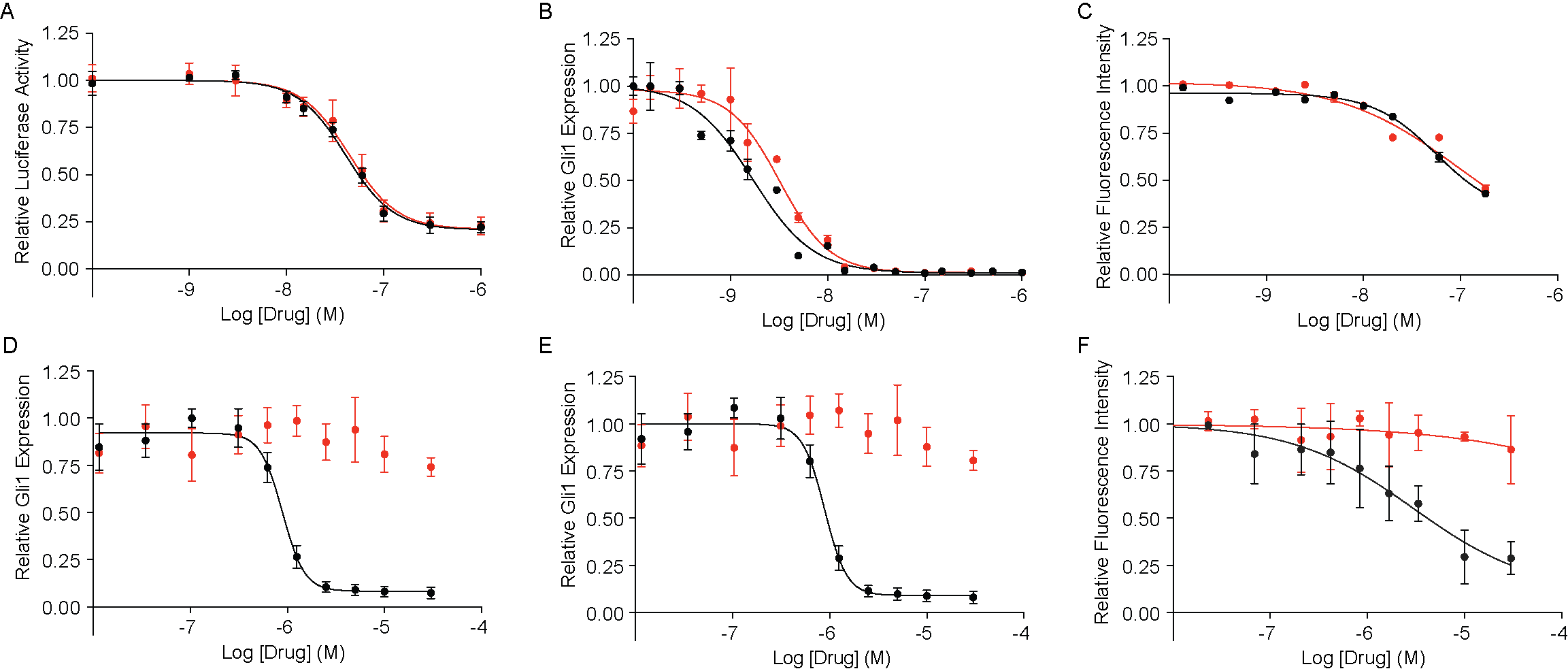

Supplement: S3 Fig — Itraconazole inhibits Smo via an aggregation-based mechanism Gli-luciferase reporter activity in Ptch1-/- MEFs (left), qPCR of Gli1 transcript in Ptch1-/- MEFs (middle), and direct displacement of bodipy-cyclopamine (right)—(A)-(C) Effect of centrifugation on vismodegib: vismodegib antagonism of Smo is unaffected by a 20 min centrifugation of the antagonist (red) compared to control (black). (D)-(F) Effect of centrifugation on itraconazole: itraconazole activity is largely or entirely eliminated by a 20 min centrifugation of the antagonist (red) compared to control (black). Error bars: standard deviation, combined replicates n = 3. (TIF) [file pone.0160365.s003.tif]
