# Supplementary material for: Identification of Novel Smoothened Ligands Using Structure-Based Docking
Source: PLoS One. 2016 Aug 4;11(8):e0160365. doi: 10.1371/journal.pone.0160365 (PMC4973902; doi:10.1371/journal.pone.0160365)
Supplement: S1 Table — (PDF) [file pone.0160365.s004.pdf]

S1 Table Results of first screen

Rank	Compound	Inhibition of Gli-Luciferase	IC_{50} μM (range, n)	Inhibition - Gli1 qPCR	IC_{50} μM (range, n)	Binding	IC_{50} μM (range, n)
**3**	C55270268	yes	43.0 (28.3-63.0, 4)	yes	94.8 (72.0-124.9, 3)	yes	166 (130-212, 2)
**6**	C48326431	yes	20.8 (12.7-34.2, 3)	yes	109 (50.3-237.8, 3)		
**15**	C25216461	no		no			
**22**	C67090834	no					
**42**	C66303859	no					
**44**	C72143438	yes	34.4 (7.6-155, 3)	yes	14.0 (11.4-17.3, 2)	yes	15.6 (8.2-3.0, 3)
**45**	C08112633	no					
**62**	C71853530	no					
**77**	C69810396	no					
**79**	C67472686	no					
**96**	C44950101	no					
**109**	C69457212	no					
**120**	C31712840	no					
**164**	C58316861	no					
**197**	C66148997	no					
**230**	C79031196	no					
**244**	C72431875	yes	5.3 (3.0-9.5, 3)	yes	11.3 (2.2-58.1, 1)	yes	58.3 (26.2-130, 1)
**265**	C82230248	no					
**278**	C12802040	no					
**377**	C11697764	no					
**427**	C77929373	no					
