# Supplementary material for: Identification of Novel Smoothened Ligands Using Structure-Based Docking
Source: PLoS One. 2016 Aug 4;11(8):e0160365. doi: 10.1371/journal.pone.0160365 (PMC4973902; doi:10.1371/journal.pone.0160365)
Supplement: S2 Table — (PDF) [file pone.0160365.s005.pdf]

S2 Table Results of analog screen

Compound number	Rank	Compound	Inhibition of Gli-Luciferase	IC_{50} μM (range, n)	Inhibition - mGli1 qPCR	IC_{50} μM (range, n)	Binding	IC_{50} μM (range, n)
**1b**	61	C72129543	*aggregator**					
**2b**	43	C72146912	no					
**3b**	48	C72158134	yes				yes	
**4b**	28	C72167017	yes				yes	
**5b**	53	C72168261	yes				yes	
**6b**	41	C72172828	no					
**7b**	52	C72419993	yes				yes	
**8b**	4	C72426960	yes					
**9b**	178	C72481543	yes					
**10b**	33	C72431634	no					
**11b**	11	C72473625	yes					
**12b**	68	C72429353	yes				yes	
**13b**	N/A	C55271488	yes	10.9 (8.2-14.5, 3)				
**14b**	2	C72129787	yes					
**15b**	18	C72149026	no				yes	
**16b**	12	C72149186	yes					
**17b**	20	C72149758	yes					
**18b**	229	C72150023	no				no	
**19b**	5	C72150480	yes	22.4 (5.7-87.2, 3)				
**20b**	9	C72153124	*aggregator**					
**21b**	6	C72158119	yes	bell-shape (3)				
**22b**	13	C72163209	yes					
**23b**	36	C72163710	no				yes	
**24b**	15	C72168023	yes	bell-shape (3)				
**25b**	34	C72168579	yes	2.3 (1.5-3.6, 3)	yes	7.8 (4.0-15.1, 3)		
**26b**	3	C72408285	yes					
**27b**	1	C72420973	*aggregator**					
**28b**	228	C72435718	no				yes	
**29b**	107	C72447326	yes					
**30b**	10	C72448241	yes					
**31b**	227	C72457741	yes				no	
**32b**	224	C72477710	yes	9.4 (5.8-15.0, 3)	yes	12.7 (3.8-42.3, 3)	yes	
**33b**	19	C72476169	yes					
**34b**	35	C72480679	yes					
**35b**	159	C72428267	no					
**36b**	96	C72433192	no					
**37b**	54	C72146027	yes	5.4 (3.8-7.6, 3)	yes	8.8 (5.2-14.9, 3)		
**38b**	231	C72170378	no					
**39b**	204	C72152697	no					
**40b**	114	C72167102	*aggregator**					
**41b**	126	C72479818	no					
**42b**	186	C72162059	no					
**43b**	31	C72447879	yes	13.3 (5.7-31.2, 3)	yes			
**44b**	185	C72163442	yes					
**45b**	76	C72475536	yes	3.1 (1.9-5.1, 3)	yes	5.3 (3.8-7.5, 3)	yes	12.7 (9.4-17.3, 3)
**46b**	230	C72447458	yes	15.8 (9.0-27.8, 3)	yes			
		vismodegib (nM)	yes	30.6 (12.3-76.3, 3)	yes	1.8 (1.4-2.2, 3)	yes	21.0 (13.3-33.3)

**aggregator: see S3 Table*
