# Supplementary material for: Identification of Novel Smoothened Ligands Using Structure-Based Docking
Source: PLoS One. 2016 Aug 4;11(8):e0160365. doi: 10.1371/journal.pone.0160365 (PMC4973902; doi:10.1371/journal.pone.0160365)
Supplement: S3 Table — (PDF) [file pone.0160365.s006.pdf]

S3 Table Compounds Positive in Aggregation Counter Screen

Compound	Compound number	DLS KPi	DLS DMEM	AmpC KPi	AmpC DMEM	MDH KPi	MDH DMEM
		Conc. tested (μM)		% enzyme activity**			
**C72129543**	1b	30	NA	11	NA	11	NA
**C72153124**	20b	0.8*	5*	82	86	10	17
**C72420973**	27b	0.8*	5*	15	2.5	2	4.5
**C72167102**	40b	NA	5	60		89	

*Compounds were not tested at lower concentrations.
**Compounds were tested at 100 μM.
