# Supplementary material for: Identification of Novel Smoothened Ligands Using Structure-Based Docking
Source: PLoS One. 2016 Aug 4;11(8):e0160365. doi: 10.1371/journal.pone.0160365 (PMC4973902; doi:10.1371/journal.pone.0160365)
Supplement: S4 Table — (PDF) [file pone.0160365.s007.pdf]

S4 Table Library Bias

Receptor (Max IC_{50})	Number of known ligands in CHEMBL19	Number of purchasable known ligands	Number of purchasable analogs of known ligands *
SMO (1 μM)	363	25	2835
ADRB2 (1 μM)	1432	206	21581
HTR2A (1 μM)	3570	397	25390
DRD2 (1 μM)	4939	469	26137

*ECFP4 cut-off = 0.8

CHEMBL 19 (Bento *et al.*, 2014; Gaulton *et al.*, 2012)
